# Dual-Target SPG-Mandibular Neuromodulation for Post-Radiation Painful Trigeminal Neuropathy: A 7-Year Follow-up

**DOI:** 10.1097/PR9.0000000000001419

**Published:** 2026-02-17

**Authors:** Vadim Tashlykov, Ofir Morag, Gabriel Lichtenshtein, Itay Goor-Aryeh, Evgeni Brotfain

**Affiliations:** a Pain Clinic, Department of Anesthesiology, Samson University Medical Center, Public Hospital Assuta, Ashdod, Israel. Ben-Gurion University of the Negev, Beer Sheva, Israel; b Department of Anesthesiology, Pain Institute, Sheba Medical CenterTel-HaShomer, Ramat Gan, Israel,; c Division of Anesthesiology and Critical Care, Soroka Medical Center, Faculty of Health Science, Ben-Gurion University of the Negev, Beer Sheva, Israel

**Keywords:** Trigeminal neuropathy, Sphenopalatine ganglion, Peripheral nerve stimulation, Neuromodulation, Facial pain, Post-radiation pain

## Abstract

**Introduction:**

Chronic facial neuropathic pain following surgical or radiation-induced trigeminal nerve damage presents significant therapeutic challenges.

**Objectives:**

We aim to demonstrate the effectiveness of combined SPG and V3 neuromodulation as a potential therapiutic measure for trigeminal neuropathy.

**Methods:**

First, trial SPG neuromodulation was implanted. Based on significant decreaze in VAS score after trial period, the permanent SPG and, in addition, mandibular electrode (V3) were provided.

**Results:**

A 72-year-old male with diabetes mellitus and dyslipidemia presented to our pain clinic in 2017 with chronic facial pain. In 2008, he underwent left parotid gland tumor resection, chemotherapy and radiation therapy. Facial pain developed post-treatment and significantly worsened following left carotid artery stenting in 2015. Pain was localized to the left maxillary (V2) and mandibular (V3) divisions of the trigeminal nerve with constant burning pain (VAS 5/10) and superimposed electric shock-like attacks (VAS 8-9/10). After failure of multimodal pharmacological and interventional treatments, combined SPG and V3 peripheral nerve stimulation was performed. During interventional procedure, at first, permanent SPG neuromodulation and then, the mandibular electrode was introduced from the same incision. The patient achieved 90% pain reduction initially, with sustained benefit at 7-year follow-up despite some habituation.

**Conclusion:**

Combined sphenopalatine ganglion and V3 neuromodulation represents a viable therapeutic option for refractory facial neuropathic pain, particularly in cases with multiterritorial involvement.

## Abstract 2

We presented an interesting clinical case of combined sphenopalatine ganglion and V3 peripheral nerve stimulation. It demonstrates an effective treatment option for refractory facial neuropathic pain.

## 1. Introduction

The incidence of trigeminal neuralgia is about 4 to 5 per 100000 persons per year. However, there is no clear and evidence data regarding incidences of trigeminal neuropathy in the recent published literature because of heterogeneity in definitions, population, and methods. Some publications report prevalence in clinic populations ranging from 0.5% to 12%.^22^ Trigeminal neuropathy (TN) manifests in 1 or more territories of trigeminal nerve innervation (V1-ophthalmic, V2-maxillary, and V3-mandibular) and is characterized by constant, dull, burning pain with associated numbness or tingling, in opposite to trigeminal neuralgy characterized by short lasting sudden electric or sharp pain attacks.^9,22^ The sphenopalatine ganglion (SPG) is a parasympathetic ganglion in the pterygopalatine fossa. It is functionally linked to the maxillary division (V2) of the trigeminal nerve, which originates from the trigeminal (Gasserian) ganglion.^9^ TN commonly results from nerve compression by intracranial mass, facial surgery, or radiation therapy.^12^ The therapeutic approach differs significantly based on the underlying pathology and localization and includes a list of antineuropathic medications (serotoninergic, sodium-channel blockers, alpha2delta ligand blockers) and interventions,^7,13^ include peripheral nerve blocks, pulsed radiofrequency, and radiofrequency thermocoagulation of the trigeminal ganglion.^7,9,13,21^ Recently, neuromodulation technologies targeting the sphenopalatine ganglion and peripheral nerves have gained prominence.^16,18,20^ Several studies have demonstrated significant pain reduction with SPG neuromodulation (electrode implantation), showing sustained long-term benefits.^15,20,24^ Similarly, peripheral nerve stimulation of supraorbital, occipital, and infraorbital nerves has shown promising results, with at least 50% improvement in trigeminal neuralgia symptoms.^23^ We present a case of successful treatment of refractory postradiation facial neuropathic pain using combined SPG and V3 peripheral nerve stimulation.

## 2. Case report

A 72-year-old man presented to our clinic in 2017 with chronic painful trigeminal neuropathy. One year prior, he underwent parotid gland tumor (carcinoma of parotid gland, left side) resection followed by several months of chemoradiotherapy for malignancy. Subsequently, he developed constant pain that became unbearable during afternoon and evening hours. Pain was localized to the left upper neck and face in V2-V3 trigeminal distribution. The patient described severe constant burning pain (visual analog score [VAS] 5/10) with superimposed short-lasting electric shock–like attacks (VAS 8–9/10). No autonomic symptoms were present. The working diagnosis was painful trigeminal neuropathy due to surgical or radiation-induced nerve damage. Oncological follow-up with MRI imaging confirmed disease remission with no indication for additional therapy. During long-term follow-up, the patient has been treated by numerous combined pharmacological agents (opioids [fentanyl patch, oxycodone, methadone], nonopioid analgesics: nonsteroidal anti-inflammatory drugs, gabapentinoids, carbamazepine, lamotrigine, tricyclic antidepressants, serotonin-norephinephrine receptor inhibitors, atypical neuroleptics, and benzodiazepines), physical therapies (transcutaneous electrical nerve stimulation and local laser therapy), and interventional measures (trigger point injections, facial nerve blocks [supraorbital, auriculotemporal, occipital], ultrasound-guided superficial cervical plexus blocks, stellate ganglion blocks, SPG blocks, and pulsed radiofrequency). Given the refractory nature of the pain syndrome, neuromodulation was recommended. After extensive discussions, it was decided to implant an electrode for SPG stimulation. Written informed consent was obtained from the patient for the procedure and for publication of this case report.

### 2.1. Neuromodulation technique

**Figure 1.**
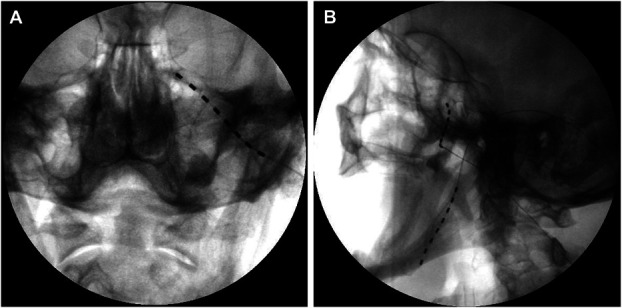
X-ray images showing sphenopalatine ganglion at Anterior-Posterior view (A) and sphenopalatine ganglion + V3 at lateral view (B) neuromodulation leads position.

Initially, a trial SPG neuromodulation was performed. During the week following implantation, the patient reported significant reduction in chronic facial pain intensity, particularly in V2 distribution. Visual analog score decreased from 7/10 to 2–3/10. However, coverage was inadequate for mandibular and upper cervical areas. Based on the significant but partial therapeutic benefit, permanent SPG neuromodulation with additional subcutaneous electrode placement in the mandibular area was performed. Both trial and permanent procedures were conducted in the operating room, with local anesthesia for the trial and general anesthesia for permanent implantation. The patient was positioned supine with head rotation to the right. The left sphenopalatine fossa was identified using C-arm fluoroscopy in lateral projection. The entry point was 1 cm anterior to the temporomandibular junction below the zygoma. Following local anesthesia, a 22-G spinal needle was used to establish the trajectory to the sphenopalatine fossa and provide deep tissue anesthesia. This was replaced by a 14-G Tuohy needle, positioned at the entrance to the sphenopalatine fossa. A metallic guide was inserted to create a pathway for electrode placement. The mandibular electrode was introduced from the same incision point to the middle of the mandible along its inferior border. Both electrodes were secured with silicone anchors and 2-0 mesh sutures to the subcutaneous tissue (Figs. 1A, B). They were tunneled subcutaneously around the ear along the sternocleidomastoid muscle to the left subclavicular area, where a subcutaneous pocket was created. The electrodes were connected to a Prodigy IPG (Abbott). Wounds were closed with 2-0 Vicryl and 3-0 nylon sutures. Programming was initiated 1 day postoperatively.

### 2.2. Postprocedural clinical outcome

Tonic stimulation of SPG created paresthesia in orbital area, base of the nose, and maxillary nerve distribution with no autonomic features in facial area. Initial programming used BURST-DR^©^ stimulation on both electrodes. The patient did not like tonic stimulation. The patient achieved 90% pain reduction immediately postprocedure, allowing discontinuation of all medications. After 3 years, early signs of habituation emerged with pain exacerbation, necessitating resumption of antineuropathic medications. At that period, continuous BURST-DR stimulation was changed by Dosing BURST-DR with 30 seconds “ON” and 90 seconds “OFF” stimulation. At 7-year follow-up, the patient continues using the implanted device with adjunctive medications (gabapentin 2400 mg/d, duloxetine 90 mg/d, and medical cannabis- THC 10%, CBD 5%, 50 gr/month), maintaining pain at VAS 2-3/10. The IPG shows 38,549 hours of use, confirming excellent long-term compliance and device utilization.

## 3. Discussion

This case demonstrates the successful application of combined SPG and V3 peripheral nerve stimulation for refractory postradiation painful trigeminal neuropathy. The dual-target approach addresses the complex innervation patterns of facial pain, particularly when involving multiple trigeminal divisions. The sphenopalatine ganglion, the largest extracranial parasympathetic ganglion, plays a crucial role in craniofacial pain processing. SPG stimulation modulates pain through several mechanisms: (1) interruption of parasympathetic outflow reducing neurogenic inflammation, (2) modulation of trigeminal nociceptive pathways through maxillary nerve stimulation and trigeminoautonomic reflex inhibition, and (3) influence on central pain processing through ascending projections to the hypothalamus and brainstem.^2,5,8^ Clinical evidence supports SPG neuromodulation efficacy, as demonstrated by Schoenen et al. who showed significant reduction in cluster headache frequency with SPG stimulation in a randomized controlled trial.^20^ Recent studies have expanded SPG applications to ischemic stroke recovery, with refined techniques showing improved outcomes through optimized electrode placement and stimulation intensity settings.^19^ Although SPG stimulation targets central pain mechanisms, peripheral nerve stimulation of trigeminal branches operates through gate control theory, activating large-diameter Aβ fibers to inhibit nociceptive transmission at the spinal trigeminal nucleus.^17^ Additionally, antidromic activation may reduce peripheral sensitization and neurogenic inflammation. Recent meta-analyses report 61.3% response rates in trigeminal neuropathic pain patients treated with peripheral nerve stimulation.^14^ The combination of SPG and peripheral V3 stimulation offers several advantages: (1) comprehensive coverage of multiple pain territories, (2) targeting both central (SPG) and peripheral (V3) pain mechanisms, and (3) potential for reduced stimulation parameters at each site, possibly delaying habituation. This multimodal neuromodulation approach aligns with current understanding of complex regional pain syndromes requiring multilevel intervention.^3^ Despite the initial success of neuromodulation therapies, habituation represents a significant challenge in long-term treatment. Defined as decreased therapeutic efficacy over time despite unchanged stimulation parameters, it affects 20% to 40% of patients within 2 to 5 years.^16^ Recent literature suggests that neural habituation parallels pharmacological tolerance, with synaptic plasticity, receptor downregulation, and fibrosis around electrodes as contributing mechanisms.^25^ Strategies to minimize habituation include (1) cycling stimulation patterns, (2) using novel waveforms like burst stimulation, (3) dosing stimulation and/or periodic “stimulation holidays,” and (4) combination with pharmacological agents targeting different pain mechanisms.^4,25^ In our patient, medication requirements remained significantly lower than preimplantation. Reprogramming successfully addressed the habituation effect, confirming continued neuromodulation efficacy. The sustained benefit in our patient over 7 years, despite some habituation, suggests that combined neuromodulation with appropriate management strategies can provide long-term efficacy. Radiation-induced neuropathy typically appears months to years after treatment and is often progressive and irreversible.^1^ Traditional treatments often fail due to the complex neuropathic mechanisms involved, including nerve compression by radiation-induced fibrosis, direct axonal damage, and vascular ischemia.^10^ Also, in our experience, destruction of Gasserian ganglion in cases of trigeminal neuropathy is less effective than treatment of classical trigeminal neuralgia with higher rate of anesthesia dolorosa complications. Previous reports of combined neuromodulation for facial pain are scarce. Recent case reports have shown promising results with peripheral nerve stimulation for postradiation trigeminal neuropathy, although long-term outcomes remain limited.^6,11,23^

This case report has inherent limitations that must be acknowledged. As a single case, generalizability is limited. The lack of a control comparison and potential placebo effects cannot be excluded. Long-term follow-up beyond 7 years would provide additional insights into durability and late complications. Also, long-term success requires vigilant follow-up, programming optimization, and integration with multimodal pain management strategies.

## 4. Conclusion

Combined SPG and V3 peripheral nerve stimulation represents our experience in treatment such dilemmatic population of patients involving multiple trigeminal territories or postradiation etiology. Further prospective studies are warranted to establish optimal patient selection criteria and stimulation parameters for combined neuromodulation approaches in facial pain syndromes.

## Disclosures

Dr. Vadim Tashlykov is part of the teaching staff for Medtronic, Abbott, and Boston Scientific. The authors have no conflicts of interest to declare.
